# Development of a Diagnostic Questionnaire for Damp Phlegm Pattern and Blood Stasis Pattern in Coronary Heart Disease Patients (CHD-DPBSPQ)

**DOI:** 10.1155/2019/6856085

**Published:** 2019-11-26

**Authors:** Ge Fang, Ling-lin Zhang, Qi Ren, Xiao-wen Zhou, Bin Wang, Xuan Zhou, Xiao-qi Liu, Dan-hong Peng, Xin-lin Chen, Xian-tao Li

**Affiliations:** School of Basic Medical Science, Guangzhou University of Chinese Medicine, Guangzhou, Guangdong Province 510006, China

## Abstract

**Background:**

The aim was to develop a diagnostic questionnaire for damp phlegm pattern and blood stasis pattern in coronary heart disease patients (CHD-DPBSPQ).

**Methods:**

The standard procedures of questionnaire development were carried out to develop and assess CHD-DPBSPQ. The patients were assessed using the CHD-DPBSPQ, CHD-DPPQ, and CHD-BSPQ. Four methods were used to select the items on the CHD-DPBSPQ in a pilot study based on data from a Guizhou tertiary grade A hospital. Cronbach's alpha and the split-half reliability, test-retest reliability, content validity, criterion validity, construct validity, and convergent validity were determined in a validation study using a nationwide sample.

**Results:**

After item selection, the CHD-DPBSPQ contained 15 items in two domains: the phlegm domain (9 items) and the blood stasis domain (6 items). For the CHD-DPBSPQ, the alpha coefficient was 0.88, the split-half coefficient was 0.90, and the intraclass correlation coefficient was 0.83. The range of the item-level content validity index (I-CVI) was 0.71 to 1.0 and that of the scale-level content validity index/average (Scale-CVI/Ave) was 0.97. The domain scores on the CHD-DPBSPQ were in close relation to the scores on a questionnaire for damp phlegm pattern in coronary heart disease patients (CHD-DPPQ) and a questionnaire for blood stasis pattern in coronary heart disease patient (CHD-BSPQ) (*P* < 0.01). The root mean square error of approximation (RMSEA) was equal to 0.05 (90% CI: 0.044, 0.059). Convergent validity was demonstrated with a moderate correlation.

**Conclusion:**

The CHD-DPBSPQ is a reliable and valid instrument.

## 1. Introduction

Currently, coronary heart disease (CHD) is the most common type of cardiovascular disease (CVD) and is a major cause of death and disability among adults worldwide [1, 2]. CHD causes approximately one-third of all deaths in people older than 35 years in Western countries [3]. CHD still affects more than 10 million people in China, and an estimated 7.4 million people suffer from CHD every year. In particular, approximately 3 million Chinese individuals die of CHD each year; the mortality rate is second only to that of cerebrovascular disease [4, 5]. This may be related to increased serum cholesterol levels caused by smoking and dietary changes [6]. Under the influence of diabetes, hypertension, and hyperlipidaemia, the incidence of CHD increases annually.

Damp phlegm pattern and blood stasis pattern (DPBSP) is commonly found in CHD patients. Along with changes in lifestyle habits, DPBSP is becoming increasingly common [7–9]. Turbid phlegm and blood stasis are two important risk factors in the development of DPBSP. According to Chinese Medicine (CM), turbid phlegm is formed by body fluids, and its clinical manifestations include cough with sputum, chest tightness, dizziness, body fat accumulation, atherosclerosis, and hyperlipidaemia. Blood stasis is caused by illiquidity, and its clinical manifestations include tingling, localized pain, enclosed masses, dark purple tongue, and hemorheology [10]. Patients with CHD-DPBSP tend to have both phlegm and blood stasis pathological manifestations [11]. Bi et al. reported that DPBSP is found in 73.42% of CHD patients, and it is a primary syndrome in these patients [12].

TCM syndromes can be studied using measures such as scales. In recent years, some researchers have developed diagnostic questionnaires for CHD with stable angina (syndrome involving both phlegm and blood stasis) [13–15]. However, these questionnaires have some shortcomings. First, the process of developing these scales is not standardized. The method of determining construct validity is not suitable, as construct validity should be determined through confirmatory factor analysis (CFA) rather than exploratory factor analysis (EFA) [16]. Second, the items contained in these questionnaires are largely divergent; thus, the diagnosis of CHD is not systematic or consistent. For example, among the four diagnostic methods of TCM (inspection, listening and smelling, inquiry, and palpation), palpation was the main item in one diagnosis questionnaire [15], but we cannot find this aspect in others [13, 14]. Some experts insist that tongue and pulse information are also very important for the diagnosis of DPBSP and can increase the diagnostic accuracy [17–21].

Therefore, we aimed to develop and assess a damp phlegm pattern and blood stasis pattern questionnaire for patients with coronary heart disease (CHD-DPBSPQ) based on a series of standard and systematic procedures of instrument development.

## 2. Methods

To develop the CHD-DPBSP diagnostic questionnaire, we used standard procedures for developing and validating the CHD-DPBSPQ [22–29]. The procedures included the following steps: construct definition, item generation, a pilot study, and a validation study. Construct definition and item generation were used to define the structure and generate the initial items for item selection. As this methodology has been reported in other papers [30–33], it will not be reported in detail in this study. The pilot study was used to select the items for the CHD-DPBSPQ, and the validation study was applied to assess the reliability and validity of the CHD-DPBSPQ.

### 2.1. Item Source

All the items on the CHD-DPBSPQ were drafted from systematic reviews, the Delphi method and analytic hierarchy process (AHP) [31–33]. Ultimately, 20 items were generated for CHD-DPBSPQ20 [34]. According to the theory of TCM, (1) chest distress, sleepiness, physical heaviness, obesity, sticky mouth, greasy tongue fur, slippery pulse, wiry pulse, abdominal fullness, anorexia, viscous stool, and taut and slippery pulse were used to assess the phlegm pattern. (2) Chest pain, cyanotic lips, dim complexion, dark purple tongue, petechiae or ecchymosis on the tongue, sublingual vein cyanosis, uneven pulse, and taut and uneven pulse were used to assess the blood stasis pattern in CHD patients.

### 2.2. Pilot Study

#### 2.2.1. Samples

A pilot study was adopted to identify items for the CHD-DPBSPQ. Recruited from a Guizhou tertiary grade A hospital, all of the CHD patients with DPBSP provided informed consent prior to their participation. The eligibility criteria are as follows: (1) diagnosis of CHD according to the Chinese guidelines for diagnosed with CHD based on the Chinese guidelines for the diagnosis and treatment of patients with chronic stable angina published in 2007 [35]; (2) diagnosis of DPBSP by 2 experienced experts [36]; (3) ≥40 years of age; and (4) provided informed consent for participation. Patients diagnosed with other syndromes or other diseases were excluded.

#### 2.2.2. Analytical Methods

Four methods were employed to select the items for the CHD-DPBSPQ: (1) the coefficient of variation: if the standard deviation (SD) of every item was less than 0.9, the item was discarded; (2) EFA: if the correlation coefficient was less than 0.4 after factor rotation, the item was deleted; (3) alpha reduction: if Cronbach's alpha coefficient was apparently increased if one item was removed, that item was marked; and (4) correlation analysis: if the item had a proportion less than 0.4, the item was abandoned [37–39]. If an item met three or more of the abovementioned criteria, it was removed.

### 2.3. Validation Study

#### 2.3.1. National Sample

A multicentre dataset was used to assess the CHD-DPBSPQ. Patients from Guangdong Provincial Hospital of Traditional Chinese Medicine, Hunan University of Traditional Chinese Medicine, Hubei Provincial Hospital of Traditional Chinese Medicine, Affiliated Hospital of Shandong University of Traditional Chinese Medicine, Guizhou Provincial Hospital of Traditional Chinese Medicine, Second Affiliated Hospital of Wenzhou Medical University, Changzhou City Hospital of Traditional Chinese Medicine, and Tianjin University of Traditional Chinese Medicine were enrolled. All patients provided informed consent for participation. The inclusion criteria were as follows: (1) diagnosis of CHD on the basis of the Chinese guidelines for the diagnosis and treatment of patients with chronic stable angina published in 2007 [35]; (2) diagnosis of DPBSP or other syndromes by 2 experienced TCM doctors [36]; (3) ≥40 years of age; and (4) provided informed consent for participation. The exclusion criteria were as follows: (1) unstable angina; (2) age <40 years or >75 years; and (3) diagnosis of other diseases or other syndromes.

Two TCM doctors with over 20 years of experience in CVD performed the assessments, and the doctors explained the questionnaire to each patient. The questionnaire included a demographic portion, followed by the CHD-DPBSPQ, CHD-DPPQ, and CHD-BSPQ. The demographic portion collected data on age, sex, ethnicity, marital status, occupation, and other diseases. The CHD-DPPQ and CHD-BSPQ were used to evaluate the criterion validity. The CHD-DPBSPQ was used for diagnosis by two experienced TCM doctors, and these two doctors could independently differentiate between the syndromes. If their diagnoses were inconsistent, diagnosis was made by a third doctor (associate professor or higher). Terwee et al. [40] believed that more than 50 samples should be assessed to determine test-retest reliability. At least 50 patients in ward settings were assessed by applying the CHD-DPBSPQ within 1–7 days, which is an applicable period to assess test-retest reliability.

#### 2.3.2. Methods Used to Evaluate the Scale

SPSS version 22.0 and Amos software 22.0 were used for data analysis [41, 42]. This scale was evaluated using classical test theory (CTT), including reliability and validity measures.

Internal consistency reliability, split-half reliability, and test-retest reliability were all evaluated [16, 43]. The internal consistency reliability was estimated using Cronbach's *α*. After the arrangement of odd and even numbers, split-half reliability was calculated using Pearson's correlation coefficients. Test-retest reliability was assessed by adopting the intraclass correlation coefficient (ICC) and its 95% confidence interval.

Validity was evaluated as content validity, criterion validity, construct validity, and convergent validity. The content validity of the questionnaire was assessed using the item-level content validity index (I-CVI) and the scale-level content validity index (S-CVI). When more than 6 experts assessed this questionnaire, the value of the I-CVI was not less than 0.78 [44–46]. We had 7 experts assess this questionnaire, and the value of the S-CVI/Ave exceeded 0.90, which indicated a high degree of content validity. The correlation coefficients between the CHD-DPPQ and the CHD-BSPQ were calculated to assess the criterion validity. CFA was used to evaluate whether the theoretical model was suitable for the data [28]. The goodness of fit index (GFI), adjusted goodness of fit index (AGFI), comparative fit index (CFI), and normed fit index (NFI) were greater than 0.9, indicating that the model was suitable. The root mean square error of approximation (RMSEA) was 0.05, indicating that the fit was close to good [47]. The correlation coefficients of the subscales and items were equal to or greater than 0.4, suggesting good convergence validity [48].

## 3. Results

### 3.1. Pilot Study

Ultimately, 103 CHD patients with DPBSP were involved in the pilot study. The age of the patients ranged from 46 years to 91 years (mean age: 68.8, Table 1); 60.2% of the sample was male, and 91.3% of the sample were retirees. Table 1 presents a brief demographic summary of the sample. The Cronbach's alpha coefficient of the CHD-DPBSPQ20 was 0.835. The results of item selection are shown in Table 2.

Of the 20 items, 5 items were removed from the original pool based on the selection criteria (*n* ≥ 3), namely, taut and slippery pulse, viscous stool, wiry pulse, uneven pulse, and taut and uneven pulse. Thus, CHD-DPBSPQ ultimately contained 15 items in 2 domains—the phlegm syndrome domain and the blood stasis syndrome domain. (1) The phlegm syndrome domain included chest distress, sleepiness, physical heaviness, obesity, sticky mouth, abdominal fullness, anorexia, greasy tongue fur, and slippery pulse. (2) The blood stasis syndrome domain included chest pain, cyanotic lips, dim complexion, dark purple tongue, petechiae or ecchymosis on the tongue, and sublingual vein cyanosis.

### 3.2. Validation Study

To further improve the demographic composition of the sample, data were collected from the clinical population of 8 hospitals in China. The demographic characteristics of the 729 total participants are shown in Table 1. Statistically, the mean age was 67.5 ± 10.6 years (range, 32.0–91.0 years), and a total of 460 male and 269 female patients were included. Among these patients, 99.3% were of Han ethnicity, 67.5% were retirees, 99.5% were married, and 88.0% had another disease. The sample (*N* = 729) was measured for the first time, with 81 of the patients measured a second time.

Table 2 shows the item distributions, which were assessed using means, standard deviation (SD), and missing data. In total, 729 patients were enrolled and completed the questionnaire. All the item scores were between 0 and 4 (Table 3). Chest distress had the highest score (1.94), while sleepiness had the lowest (0.45). Chest pain, sticky mouth, and cyanotic lips had missing values.

#### 3.2.1. Reliability

The CHD-DPBSP had high internal consistency and retest reliability. The mean score on the CHD-DPBSPQ was 17.4, the mean score on the phlegm domain was 9.7, and the mean score on the blood stasis domain was 7.7 (Table 4). ① The Cronbach's alpha values for both domains were greater than 0.75 (Table 4). ② The split-half coefficients of the domains were greater than 0.80 (Table 4). ③ The interitem correlation coefficient (ICC) of the domains were 0.78, 0.83, and 0.83, which were more than 0.8, and the retest reliability coefficient was high (Table 4).

#### 3.2.2. Validity

**Figure 1:**
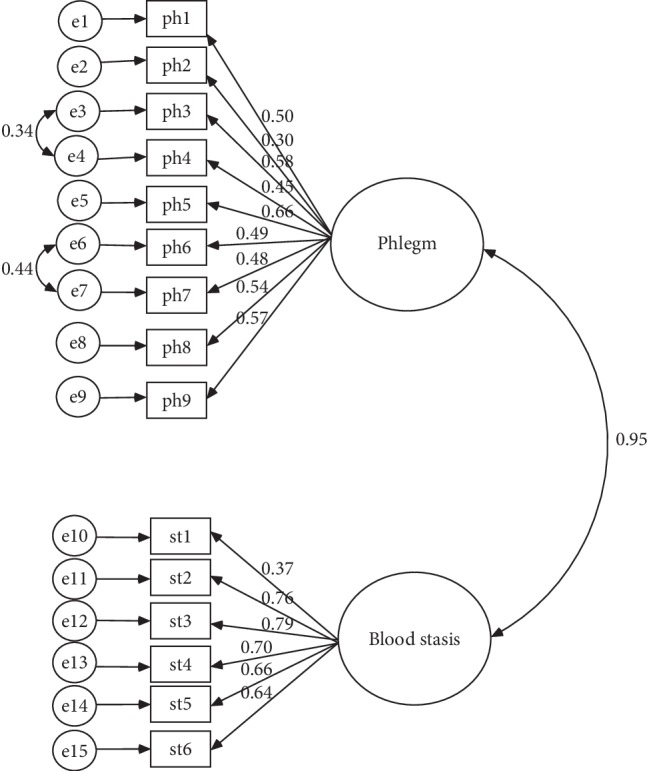
Standardized regression weight in the questionnaire confirmatory factor analysis model. ph1: chest distress, ph2: sleepiness, ph3: physical heaviness, ph4: obesity, ph5: sticky mouth, ph6: abdominal fullness, ph7: anorexia, ph8: greasy fur, and ph9: slippery pulse; st1: chest pain, st2: cyanotic lips, st3: dim complexion, st4: dark purple tongue, st5: petechiae or ecchymosis on the tongue, and st6: sublingual vein cyanosis.

The CHD-DPBSPQ had good validity. ① For content validity, the range of the I-CVI was 0.71 to 1.0, and the S-CVI/Ave was 0.97 (Table 5). ② The criterion validity was checked by comparing the scale with the CHD-DPPQ and the CHD-BSPQ. The correlation coefficient between the phlegm domain and the CHD-DPPQ was 0.76, and the correlation coefficient between the blood stasis domain and the CHD-BSPQ was 0.96 (*P* < 0.01). ③ The model fit for the scale was tested using CFA (Figure 1). The GFI, AGFI, NFI, IFI, TLI, and CFI were all greater than 0.90, and the RMSEA was 0.05 (90% CI: 0.044, 0.059) (Table 6). All these indexes indicated that the model fit was good.④ Convergent validity was demonstrated by a moderate correlation (0.423–0.796), as shown in Table 7.

## 4. Discussion

At present, the specific scales used in patients with CHD abroad are the Seattle angina scale (SAQ) [49] and the cardiovascular limitations and symptoms profile (CLASP) [50]. Nevertheless, owing to cultural differences, we researched and developed the CHD-DPBSPQ to diagnose DPBSP in Chinese CHD patients.

Previous instruments for the assessment of CHD-DPBSP have not been widely adopted; our group formulated and validated TCM outcomes on the basis of standard development and validation procedures [22, 28, 40]. The US FDA and the WHOQOL group proposed the establishment of a conceptual framework for questionnaires [23, 26]. According to TCM theories, the framework for DPBSP should be classified into damp phlegm and blood stasis patterns [10, 11]. The diagnosis of DPBSP requires the simultaneous diagnosis of sputum and blood stasis [11, 20, 51, 52]. However, these questionnaires [13–15] all contain different frameworks. For example, one questionnaire involved the division of dimensions into phlegm and blood stasis [14], but the other two did not [13, 15]. Moreover, some important validity coefficients, such as convergent validity, were not evaluated. The method of determining content validity was not suitable, as content validity should be determined according to the CVI [45], but none of the three questionnaires used the CVI [13–15]. The method of construct validity was also not suitable, as construct validity should be determined via CFA rather than EFA [16].

The 15 items on the CHD-DPBSPQ were empirically selected by four methods using a sample population from a Guizhou tertiary grade A hospital. The CHD-DPBSPQ was verified with national data, showing that it is a reliable and valid tool for research and clinical trials. (1) The internal consistency of the domains was expressed by Cronbach's *α* coefficients (0.77–0.88) and split-half reliability coefficients (0.81–0.90). Terwee et al. considered that the Cronbach's *α* showed a good internal consistency range, from 0.70 to 0.95 [40], and the CHD-DPBSPQ had good internal consistency. Moreover, eighty-one patients completed the CHD-DPBSPQ a second time, and the ICC was between 0.78 and 0.83, indicating good reproducibility [40].

(2) The content validity of the questionnaire was evaluated by seven experts. A standard value of I-CVI greater than 0.78 was recommended by Lynn [53]; the score for sticky mouth was 0.71, but the Kappa-like index (*K*) was 0.65 (>0.60), which showed good validity [54]. The S-CVI for the scale was 0.97, which meets the requirements of the standard value (0.90) recommended by Polit and Beck [55]. The correlation coefficient between the phlegm domain and the CHD-DPPQ was 0.76, and the correlation coefficient between the blood stasis domain and the CHD-BSPQ was 0.96. Their values were greater than 0.7, which indicated positive criterion validity [40]. The RMSEA was equal to 0.05 (90% CI: 0.044, 0.059), and the NFI, NNFI, and CFI were greater than 0.90. These results indicated that the scale had good construct validity [47]. The correlations of the CHD-DPBSPQ between the phlegm domain and our hypothesized components (chest distress, sleepiness, physical heaviness, obesity, sticky mouth, abdominal fullness, anorexia, greasy tongue fur, and slippery pulse) were greater than 0.4, as were the correlations of the CHD-DPBSPQ between the blood stasis domain and our hypothesized components (chest pain, cyanotic lips, dim complexion, dark purple tongue, petechiae or ecchymosis on the tongue, and sublingual vein cyanosis), indicating moderate convergence validity [48]. In short, these findings are encouraging and support the structural integrity of the CHD-DPBSPQ.

The purpose of our study is to develop and validate the CHD-DPBSPQ, a questionnaire intended for use in the clinical practice of TCM. However, the questionnaire is not applicable for diagnosing CHD and all TCM syndromes of CHD. Thus, the diagnosis of CHD was first confirmed in patients recruited for our study according to the Chinese guidelines for the diagnosis of CHD, which were based on the Chinese guidelines for the diagnosis and treatment of patients with chronic stable angina published in 2007 [35]. The CHD-DPBSPQ was developed on the basis of the theory of TCM syndromes. Meanwhile, structural equation modelling is a valid method for testing TCM syndromes, as described in other studies [23, 29]. Up to now, other researchers have formulated and exerted similar methods to explore TCM syndromes [16, 24]. For example, Chen et al. developed the LIDHS questionnaire and evaluated its reliability and validity as a “TCM Syndrome Questionnaire of Ulcerative Colitis” [16]. The CHD-DPBSPQ, as an effective and reliable pattern of TCM questionnaire, can also be used to diagnose CHD-DPBSP in patients.

There were a few limitations. (1) The patients were retested again 1–7 days after the first investigation. Thus, test-retest reliability could have been overestimated due to the short time interval. (2) No pathological data were provided for the diagnosis of CHD-DPBSP. (3) The pilot study enrolled patients from a Guizhou tertiary grade A hospital. The CHD-DPBSPQ should be further assessed throughout every province in the country. (4) The gold standard adopted was the CHD-DPPQ and CHD-BSPQ developed in China, which do not include the internationally recognized scale for CHD.

This questionnaire also has some strengths. First, compared with one published scale [13], our questionnaire was more convincing in terms of verification. Second, the development of a diagnostic CHD-DPBSPQ is rare. Third, 100% of the patients completed the questionnaire. Fourth, the content of our scale was easy to understand, and the average completion time for the questionnaire was 5.4 minutes. Finally, it was confirmed that the results were generalizable by using a multicentre clinical investigation.

## 5. Conclusions

A DPBSP questionnaire was developed for patients with CHD. The 20 items were successfully reduced to 15 items during the selection process, a quantitative stage of questionnaire development. The CHD-DPBSPQ showed good reliability and validity and is feasible for diagnosing DPBSP in CHD patients. We recommend the application of the CHD-DPBSPQ for diagnosing DPBSP in Chinese CHD patients.

## Figures and Tables

**Table 1:** Demographic characteristics of the patients with CHD-DPBSP.

Characteristic	Pilot study CHD-DPBSP (%, *n* = 103)	Validation study	
		CHD-DPBSP (%, *n* = 729)	CHD-DPBSP (%, *n* = 81)^*∗*^
Age, mean ± SD (range)	68.8 ± 9.2 (46, 91)	67.5 ± 10.6 (32.0, 91.0)	66.9 ± 12.2 (38.0, 90.0)

Sex			
Male	62 (60.2)	460 (63.1)	48 (59.3)
Female	41 (39.8)	269 (36.9)	33 (40.7)

Ethnicity			
Han	101 (98.1)	724 (99.3)	81 (100.0)
Others	2 (1.9)	5 (0.7)	0 (0)

Marital status			
Married	103 (100)	725 (99.5)	100 (100.0)
Unmarried	0 (0)	4 (0.5)	0 (0)

Occupation			
Worker	0 (0)	66 (9.1)	9 (11.1)
Farmer	3 (2.9)	73 (10.0)	7 (8.6)
Specialist	3 (2.9)	26 (3.6)	2 (2.5)
Staff	3 (3.9)	67 (9.2)	11 (13.6.0)
Retiree	94 (91.3)	497 (67.5)	52 (64.2)

Other disease			
Yes	85 (82.5)	641 (88.0)	71 (87.7)
No	18 (17.5)	88 (12.0)	10 (12.3)

CHD-DPBSP: damp phlegm pattern and blood stasis pattern for coronary heart disease. ^*∗*^These patients were used for the retest.

**Table 2:** The results of item selection (pilot study).

Items	CV	EFA	*α* value	*r*	Deleted item
Chest distress	0.531	0.605	0.670	0.642	
Chest pain	1.115	0.718	0.752	0.757	
Sleepiness	1.100	0.286	0.689	0.465	
Physical heaviness	0.984	0.613	0.658	0.625	
Obesity	1.237	0.506	0.669	0.587	
Sticky mouth	1.171	0.752	0.665	0.594	
Cyanotic lips	0.725	0.740	0.747	0.769	
Dim complexion	0.999	0.849	0.712	0.881	
Abdominal fullness	1.066	0.782	0.632	0.748	
Anorexia	1.075	0.717	0.645	0.686	
Viscous stool	0.651	-0.180	0.710	0.183	×
Dark purple tongue	0.666	0.773	0.747	0.785	
Petechiae or ecchymosis on the tongue	1.290	0.859	0.730	0.849	
Sublingual vein cyanosis	0.815	0.782	0.729	0.843	
Greasy tongue fur	0.847	0.779	0.644	0.730	
Slippery pulse	0.976	0.729	0.642	0.713	
Wiry pulse	1.031	−0.485	0.749	0.026	×
Uneven pulse	0.429	−0.328	0.833	−0.147	×
Taut and slippery pulse	1.240	−0.503	0.771	−0.003	×
Taut and uneven pulse	0.589	−0.350	0.846	−0.113	×

CV: coefficient of variation; EFA: exploratory factor analysis; *α* value: alpha reduction; *r*: correlation analysis. If the item met three or more of the abovementioned criteria, it was deleted.

**Table 3:** Lacking data, mean, and SD for each item (*n* = 729).

Item	Score 0	Score 1	Score 2	Score 3	Lacking	Mean	SD
Chest distress	99	116	245	269	0	1.94	1.03
Chest pain	247	141	190	150	1	1.33	1.15
Sleepiness	527	102	71	29	0	0.45	0.83
Physical heaviness	316	127	236	50	0	1.03	1.02
Obesity	325	142	206	56	0	0.99	1.02
Sticky mouth	348	164	137	79	1	0.93	1.05
Cyanotic lips	269	189	167	103	1	1.14	1.07
Dim complexion	275	179	166	109	0	1.15	1.09
Abdominal fullness	414	115	151	49	0	0.77	1
Anorexia	429	132	127	41	0	0.7	0.95
Dark purple tongue	196	125	264	144	0	1.49	1.09
Petechiae or ecchymosis on the tongue	297	130	167	135	0	1.19	1.16
Sublingual vein cyanosis	191	188	214	136	0	1.4	1.07
Greasy tongue fur	120	158	302	149	0	1.66	0.98
Slippery pulse	308	93	191	137	0	1.22	1.18

SD: standard deviation.

**Table 4:** Descriptive statistical data and reliability of the CHD-DPBSPQ.

	No. of items	Mean ± SD	Range of score	Cronbach's alpha	Split-half coefficient	ICC (95% CI)
Phlegm domain	9	9.7 ± 5.4	(0.0, 27.0)	0.77	0.81	0.78 (0.66, 0.86)
Blood stasis domain	6	7.7 ± 4.7	(0.0, 18.0)	0.81	0.81	0.83 (0.74, 0.89)
CHD-DPBSPQ	15	17.4 ± 9.5	(0.0, 45.0)	0.88	0.90	0.83 (0.74, 0.89)

ICC: intraclass correlation coefficient.

**Table 5:** The values of I-CVI for each item and S-CVI/Ave for the questionnaire.

Item	I-CVI	No. of respondents
Chest distress	1.00	7
Chest pain	1.00	7
Sleepiness	1.00	7
Physical heaviness	1.00	7
Obesity	1.00	7
Sticky mouth	0.71	7
Cyanotic lips	1.00	7
Dim complexion	0.86	7
Abdominal fullness	1.00	7
Anorexia	1.00	7
Dark purple tongue	1.00	7
Petechiae or ecchymosis on the tongue	1.00	7
Sublingual vein cyanosis	1.00	7
Greasy tongue fur	1.00	7
Slippery pulse	1.00	7
S-CVI/Ave	0.97^*∗*^	7

I-CVI: item-level content validity index. ^*∗*^: scale-level content validity index, scale-level CVI; average (scale–CVI/Ave).

**Table 6:** Model fit test results for the CFA model.

Models	*χ* ^2^/d*f*	RMSEA	GFI	AGFI	NFI	IFI	TLI	CFI
2-factor model	2.90	0.05	0.96	0.94	0.93	0.95	0.94	0.95

CFA: confirmatory factor analysis, RMSEA: root mean square error of approximation, GFI: goodness of fit index, AGFI: adjusted goodness of ft index, NFI: normed fit index, IFI: increment fitting index, TLI: Turker-Lewis index, CFI: comparative fit index.

**Table 7:** Correlation coefficients between each item and different domains.

	Phlegm domain	Blood stasis domain
Phlegm		
Chest distress	0.571^*∗∗*^	0.435
Sleepiness	0.423^*∗∗*^	0.244
Physical heaviness	0.687^*∗∗*^	0.509
Obesity	0.569^*∗∗*^	0.411
Sticky mouth	0.695^*∗∗*^	0.569
Abdominal fullness	0.625^*∗∗*^	0.423
Anorexia	0.586^*∗∗*^	0.432
Greasy tongue fur	0.584^*∗∗*^	0.472
Slippery pulse	0.608^*∗∗*^	0.507

Blood stasis		
Chest pain	0.345	0.538^*∗∗*^
Cyanotic lips	0.580	0.786^*∗∗*^
Dim complexion	0.642	0.796^*∗∗*^
Dark purple tongue	0.564	0.748^*∗∗*^
Petechiae or ecchymosis on the tongue	0.559	0.734^*∗∗*^
Sublingual vein cyanosis	0.556	0.706^*∗∗*^

^*∗∗*^ *P* value <0.01.

## Data Availability

The data used to support the findings of this study are available from the corresponding author upon request.

## Abbreviations

CHD-DPBSPQ: Questionnaire of damp phlegm pattern and blood stasis pattern for coronary heart disease
CVI: Content validity index
I-CVI: Item-level content validity index
S-CVI: Scale-level content validity index
CHD-DPPQ: Questionnaire of damp phlegm pattern for coronary heart disease
CHD-BSPQ: Questionnaire of blood stasis syndrome for coronary heart disease
CM: Chinese medicine
CFA: Confirmatory factor analysis
QOL: Quality of life
SD: Standard deviation
CTT: Classical test theory
ICC: Intraclass correlation coefficient
RMSEA: Root mean square error of approximation
DPBSPQ: Damp phlegm pattern and blood stasis pattern
GFI: Goodness of fit index
AGFI: Adjusted goodness of fit index
CFI: Comparative fit index
NFI: Normed fit index
RMSEA: Root mean square error of approximation
SAQ: Seattle angina scale
CLASP: Cardiovascular limitations and symptoms profile.
